# The Hip-Joint

**Published:** 1877-06

**Authors:** E. R. Barnes


ART. II.—*The Hip Joint. Paper read before the Buffalo Medical Association, May* 1st, 1877, by E. R. BARNES, M. D.

In the last paper read by me before this Association, after reviewing the anatomy of the hip joint, the subject of fracture of the neck of the femur was taken up and the points alluded to in this connection were first, the means which nature provides to guard against such accidents, and the manner in which such accidents generally occur; secondly, the periods of life within which such lesions are limited, and during which there is a greater or less liability to such injuries; thirdly, the relative frequency of extra and intra capsular fracture at different ages, with some remarks as to diagnosis between these two forms of fracture; fourthly, the kinds of union which take place after fracture of the neck; fifthly, the symptoms of this fracture, with such explanation as could be briefly presented, of the causes of these symptoms; and lastly, a reference to the treatment as derived from the previous considerations.

Before passing to the subject of the dislocation of the hip joint, I wish to call attention to a condition which closely simulates some forms of fracture. A severe blow, which does not produce a fracture, may occasion much pain and tenderness about the joint, with temporary muscular paralysis, and inflammatory action may be induced which will result in interstitial absorption of the neck of the bone, and ultimately, shortening and perhaps eversion of the limb. Chronic rheumatic arthritis may induce similar changes, but the history of the case would distinguish this condition. Like changes may also take place after a limb, which has been dislocated, has been reduced, so that shortening may ensue, although the head of the femur may have been restored to its proper place. Nature has provided against dislocation of the hip joint by the deep socket in which the head of the bone rests, and by the powerful ligaments and muscles which combine to hold the bone in place. Yet, owing to the variety of movements of which the joint is capable, the weight to which it is or may be subjected, and the powerful leverage afforded by the shaft of the thigh bone, or by the extended limb, this joint more frequently suffers dislocation than any other excepting that at the shoulder.

Dislocation of this joint is nearly always occasioned by great force. It may occur at any age, and has even been known to result from causes acting during intra-uterine life. Where there is no period between infancy and extreme age within which it has not been known to occur, yet, as might be expected, from the statement that this accident is usually produced by great force, we find that its period of greatest frequency is during the most active time of life, that is between twenty and forty-five years of age, and that it is much more common in the male than in the female. In these respects there is a marked difference between fracture and dislocation.

The position of the limb is also an important factor in supplying the conditions necessary to produce this lesion. More or less flexion of the thigh upon the pelvis, together with powerful adduction of the limb, are probably conditions precedent to the production of the upward and backward dislocations, and if we add to this, inversion, we have all the conditions under which a strong force, acting through a powerful leverage, may pry the head of the femur from its socket, causing it to rupture the ligamentum tires and the posterior portion of the capsule, and to emerge into a new and abnormal position. These conditions being induced by a fall from a height upon the floor, or upon the outer side of the knee. The dislocation upon the dorsum ilii, may occur, and that commonly described as into the ischiatic notch. If the thigh is at right angles to the pelvis, and the limb adducted when the force is applied, the head of the bone may pass directly backwards. When flexion of the thigh is carried upwards beyond a right angle, the head of the femur tends to make its exit more and more in a direction downwards and backwards towards the tuberosity of the ischium, which tendency is much favored by the simultaneous inward rotation or inversion of the limb.

Forcible abduction of the limb causes downward dislocation, whether the limb be at the same time flexed or extended. Outward rotation occurring simultaneously, greatly facilitates the escape of the head in a position of abduction, so that a slight force has been known to produce such escape, as in the case of a person stepping from a sleigh, whose foot was caught upon the edge of the vehicle and retained there until the other foot rested upon the ground, thus causing sudden but not very forcible abduction, and outward rotation of the limb, and consequent downward dislocation of the head.

We thus see that with flexion, adduction and inward rotation, the proper force being applied, the head may emerge anywhere in the circuit, from a point well up on the dorsum to the neighborhood of the tuberosity of the ischium, while with abduction, and outward rotation, the luxation downwards only is produced. These statements apply to the three dislocations included in the usual classification, as upon the dorsum, into the ischiatic notch, and into the thyroid foramen.

The mechanism of the dislocation onto the pubes is somewhat different. It is tersely described by Hamilton as being caused "by a fall upon the foot when the leg is thrown backwards behind the center of gravity;" or in other words, by a force acting from below upwards, and from behind forward, the body advancing, while the point of contact, at the foot or knee, remains stationary, or so to speak "inclines forcibly backwards," so that a powerful leverage is brought to bear to force the head forwards out of its socket, and upwards into the pubes.

Such in general are the conditions under which dislocation of the thigh may be produced, according to most authorities; either during flexion, adduction, and often inversion of the thigh, or during abduction, with perhaps outward rotation. It is highly probable, however, that the great majority of these displacements take place primarily in a direction downwards, or downwards and backwards, and that the position in which the head of the bone ultimately rests is a secondary one, being caused by the head of the bone ascending around the rim of the acetabulum, as the limb tends to resume a straight position.

It is evident, from the points referred to in this and the preceding paper, that a consideration of the age of the patient, the direction in which the force acted, and the degree of force exerted, may be of value as furnishing presumptive evidence in making a diagnosis between a dislocation of the femur at the hip, and a fracture of the neck of this bone.

It is not a part of the purpose of this paper to enter a detail of the symptoms of the different forms of dislocation, but rather to consider them as a whole, and as due to a uniform set of mechanical conditions. Sufficient to say briefly, that greater or less flexion of the thigh upon the pelvis accompanies all forms of disloca-tion almost without exception; that adduction is characteristic of the upward and backward or dorsal dislocations, while abduction accompanies the upward and forward or pubic, and generally the downward or thyroid luxations.

Inversion exists in those cases where there is adduction, and eversion in those where there is abduction. Shortening exists in all cases, except in some of the downward displacements in which there is lengthening. An unnatural fixedness of the limb, or an insuperable passive resistence to motion in certain directions, is a characteristic of all these dislocations. When the displacement is in certain directions, as upon the pubes or upon the dorsum, the head of the bone may be felt in its abnormal position, especially upon rotating the limb. When the dislocation is upon the dorsum ilium, it is well to remember not to mistake the trochanter, rendered prominent after fracture of the neck of the femur, with shortening, of the head of the bone, a misapprehension which might readily lead to error.

We have thus far attempted to state briefly the mechanical conditions under which dislocations of the hip take place, and to give a general summary of the symptoms which accompany most of these dislocations. The next step is to consider the mechanical causes of these symptoms, and the use which may be made of such knowledge to facilitate the restoration of the displaced bone by manipulation. It is needless to state that I shall follow Dr. Bigelow in what little I shall have to say on this branch of the subject, as no other writer has so clearly illustrated, and I have prepared a cadaver on which to demonstrate the general statements already made, and those to be made.

Opinions have differed much as to whether it was to the muscles, or to the ligaments that the phenomena of dislocation were due, and as to which of these elements offered the greatest resistence to reduction. Experiments, and the introduction of anæsthetics, have contributed to the solution of this question. But omitting all historical allusion to the steps which have led to the views now generally held, it may be regarded as established that the ilio femoral ligament is the essential element in controlling the phenomena of the regular dislocations, and that an intelligent knowledge of its action must guide efforts at reduction by manipulation. Dissections of cases in which dislocation has occurred, have shown that in the great majority of hip dislocations, the ilio femoral ligament remains unbroken, whatever other structures may have yielded, and that where this ligament has been torn, the characteristic features of this lesion have been lost or imperfectly presented (hence Bigelow classifies those as irregular dislocations), while on the other hand, it may be demonstrated on the cadaver that after the muscles and the remaining portion of the capsular ligament have been removed, the ilio femoral ligament alone is capable of producing the essential phenomena of the regular dislocations. If besides, the tendon of the obturation internus is allowed to remain, a demonstration of the relations of the head and neck of the femur to this tendon in some forms of dislocation may also be made. But while the action of the muscles and of untorn portions of the capsule exerts a modifying influence, and may interpose obstacles to reduction, such action is accessory and of secondary importance, so far as reduction by manipulation is concerned, provided the ilio femoral ligament is unbroken. Looking at the cadaver, we see that when the limb is extended, the branches of the ilio femoral ligament are rendered tense, and "form an unyielding suspensory band by which the femur is forcibly retained in its socket." If the limb is flexed, say at an angle of 45°, this ligament is relaxed and, the necessary conditions supplied, i. e. adduction, perhaps inversion, and force, the head slips over the acetabular rim, and seeks such a position on the dorsum as is favored by the conformation of the parts onto which it is thrown. But the ilio femoral ligament is quickly rendered tense again by this change in position, and arrests any further backward movement of the base of the neck. The consequence is that the shaft of the femur, as we see, is thrown into a position of adduction, flexion and inversion. The head of the bone is now in a position from which it can be moved in a circular direction outside of the acetabulum, from a point well up on the dorsum, around to the thyroid foranum, by flexing, circumducting and rotating the shaft of the femur in a direction opposite to that in which it is intended that the head shall move, the base of the neck being compelled to move in the segment of a circle, of which the ilio femoral ligament is the radius, and the anterior inferior spinous process of the ilium is the center. Dr. Bigelow explains this movement in a little different language when he says that the "Y. ligamenter in firmly attaching to the base of the neck of the femur the inferior spinous process of the ilium, forms a fulcrum or pivot round which the shaft and the neck of the femur can be made to revolve like opposite spokes of a wheel." The head of the bone, as stated, can thus be made to pass around from the dorsum to the thyroid foramen, and by reversing the direction of the movement of circumduction, to pass again from the latter place to the former. The positions assumed by the limb in different stages of its progress will correspond to the symptoms of the different dislocations within this range, and similarly the symptoms of dislocation upon the pubes may be reproduced with the aid of the ilio femoral ligament alone.

It is by an intelligent consideration of the action of this ligament, that our manipulations for reduction of the regular dislocations, are to be mainly controlled.

We will briefly allude to some points as to dislocations upon the dorsum. Dr. Bigelow classes as dorsal dislocations, all those which occur within the range allowed by the "Y." ligament, from that in which the head emerges on the dorsum ilii above the obturator internus, and even higher, beneath the pyriformis, to that in which the head of the bone corresponds to the lower part of the ischiatic notch. The dislocations above the obturator internus may be primary or secondary, that is, in the first case the head may originally emerge above the obturator internus tendon, leaving that unruptured, or may be converted from a lower dislocation into the higher one, with often rupture of the small rotator muscles, including the obturator internus. This author altogether discards the usual classification which includes dislocation upon or into the ischiatic notch. He says: "I believe that no dislocation upon the ischiatic notch is worthy of the name, that no satisfactory or practical result can be based upon this dislocation alone, and that it is also an error to suppose that during reduction the femur ever notably slips into the sciatic notch, or that the sciatic notch ever offers any obstacle to reduction." He thinks that the reason why this classification has been accorded prominence, is because unusual difficulty of reduction has been experienced when the head has been near this locality, and this difficulty has been ascribed to the head being engaged in the ischiatic notch, and that many observers finding reduction more difficult than usual, have reported cases as into the sciatic notch, which were in reality simple dorsal dislocations. He believes that the difficulties of reduction by the pulleys are due to other causes, either to the head of the femur emerging primarily above the obturator internus, leaving that unruptured, or to the head, in the act of ascending from below the tendon onto the dorsum, becoming by a movement of inversion engaged behind the tendon of that muscle, and the subjacent capsule, so that these interpose to prevent the return of the head into the socket. He therefore proposes a practical classification of dorsal dislocations, of some value in reference to reduction, viz.: first, that in which the head of the femur is engaged between the rotator muscles; and second, that in which the head is eugaged behind the obturator internus tendon. In the second class of dorsal dislocations, the flexion method is sometimes absolutely required to accomplish reduction.

